# Carbohydrates from Sources with a Higher Glycemic Index during Adolescence: Is Evening Rather than Morning Intake Relevant for Risk Markers of Type 2 Diabetes in Young Adulthood?

**DOI:** 10.3390/nu9060591

**Published:** 2017-06-10

**Authors:** Tanja Diederichs, Christian Herder, Sarah Roßbach, Michael Roden, Stefan A. Wudy, Ute Nöthlings, Ute Alexy, Anette E. Buyken

**Affiliations:** 1 IEL-Nutritional Epidemiology, DONALD Study, Rheinische Friedrich-Wilhelms-University Bonn, Heinstueck 11, 44225 Dortmund, Germany; tdiederi@uni-bonn.de (T.D.); srossbac@uni-bonn.de (S.R.); noethlings@uni-bonn.de (U.N.); anette.buyken@uni-paderborn.de (A.E.B.); 2 Institute for Clinical Diabetology, German Diabetes Center, Leibniz Center for Diabetes Research at Heinrich Heine University Düsseldorf, Düsseldorf, Auf’m Hennekamp 65, 40225 Düsseldorf, Germany; christian.herder@ddz.uni-duesseldorf.de (C.H.); michael.roden@ddz.uni-duesseldorf.de (M.R.); 3 German Center for Diabetes Research (DZD), Ingolstädter Landstr. 1, 85764 München-Neuherberg, Germany; 4 Department of Endocrinology and Diabetology, Medical Faculty, Heinrich Heine University Düsseldorf, Moorenstraße 5, 40225 Düsseldorf, Germany; 5 Pediatric Endocrinology and Diabetology, Laboratory for Translational Hormone Analytics, Peptide Hormone Research Unit, Center of Child and Adolescent Medicine, Justus Liebig University Giessen, Feulgenstraße 10-12, 35392 Gießen, Germany; Stefan.Wudy@paediat.med.uni-giessen.de; 6 Institute of Nutrition, Consumption and Health, Faculty of Natural Sciences, University Paderborn, Warburger Straße 100, 33098 Paderborn, Germany

**Keywords:** glycaemic index, glycaemic load, daytime, adolescence, type 2 diabetes mellitus

## Abstract

**Background:** This study investigated whether glycemic index (GI) or glycemic load (GL) of morning or evening intake and morning or evening carbohydrate intake from low- or higher-GI food sources (low-GI-CHO, higher-GI-CHO) during adolescence are relevant for risk markers of type 2 diabetes in young adulthood. **Methods:** Analyses included DOrtmund Nutritional and Anthropometric Longitudinally Designed (DONALD) study participants who had provided at least two 3-day weighed dietary records (median: 7 records) during adolescence and one blood sample in young adulthood. Using multivariable linear regression analyses, estimated morning and evening GI, GL, low-GI-CHO (GI < 55) and higher-GI-CHO (GI ≥ 55) were related to insulin sensitivity (*N* = 252), hepatic steatosis index (HSI), fatty liver index (FLI) (both *N* = 253), and a pro-inflammatory-score (*N* = 249). **Results:** Morning intakes during adolescence were not associated with any of the adult risk markers. A higher evening GI during adolescence was related to an increased HSI in young adulthood (*p* = 0.003). A higher consumption of higher-GI-CHO in the evening was associated with lower insulin sensitivity (*p* = 0.046) and an increased HSI (*p* = 0.006), while a higher evening intake of low-GI-CHO was related to a lower HSI (*p* = 0.009). Evening intakes were not related to FLI or the pro-inflammatory-score (all *p* > 0.1). **Conclusion:** Avoidance of large amounts of carbohydrates from higher-GI sources in the evening should be considered in preventive strategies to reduce the risk of type 2 diabetes in adulthood.

## 1. Introduction

Diets low in glycemic index (GI) or glycemic load (GL) are related to a lower risk of developing type 2 diabetes mellitus [1,2]. Their preferred use has hence been advocated particularly during periods of physiological insulin resistance, such as puberty [3]. This is supported by our recent observations linking a higher dietary GI/GL during puberty to a lower insulin sensitivity and increased liver enzyme activities [4] as well as increased levels of interleukin-(IL)-6 [5] in young adulthood, i.e., metabolic markers indicating an increased risk of developing type 2 diabetes in later life [6].

Recent discussions on preventive procedures also account for chronobiological aspects of metabolism [7]. This originates from the observation that extreme circadian (circa dies (lat.) = about 24 h) misalignment—as experienced during shiftwork—enhances the risk of type 2 diabetes among adults [8]. In a subsample from a Finnish population-based study, behavioral traits towards eveningness (based on a questionnaire assessing morningness-eveningness) were linked to notably higher odds for type 2 diabetes [9]. Persons with a late chronotype (i.e., those with a preference for a delayed timing of sleep on free days, i.e., without social obligations) are at particular risk of experiencing mild, but chronic misalignment resulting from the discrepancy between their circadian clock and socially determined, fixed schedules [10]. Misalignment may extend to their dietary behavior if it does not match metabolic processes, most of which follow a circadian rhythm [7]. Hence, dietary misalignment can emerge due to a discrepancy between the biological and the social timing or result from a general mismatch of dietary intake to metabolic circadian rhythmicity, e.g., to the decrease in insulin sensitivity over the day [11,12]. It is conceivable that adolescents are vulnerable to dietary misalignment since adolescence is characterized by a pronounced “lateness” in chronotype, i.e., a preference for a delayed timing of sleep on free days [13], which may be exacerbated by the physiological insulin resistance occurring during adolescence [14]. We hence hypothesize that recurring postprandial glycemic excursions elicited by carbohydrate-containing foods with a higher GI are particularly detrimental in evening hours and will have longer-term downstream adverse effects on adult metabolic health. It is possible that these are specific to either the *relative* glycemic response to the carbohydrate-containing foods consumed in evening hours, i.e., their GI [15], to their estimated postprandial glucose and insulin responses (i.e., their GL) and/or the intake of carbohydrates from low-GI (GI < 55) or higher-GI (GI ≥ 55, thus including sources with moderate- as well as high-GI) sources (low-GI-CHO, higher-GI-CHO).

Therefore, the aim of our current analysis was to separately examine the habitual dietary GI and GL of morning and evening intake during adolescence as well as morning and evening low-GI-CHO and higher-GI-CHO intake for prospective associations with risk markers of type 2 diabetes in young adulthood. Primary outcome measures comprised established risk parameters (insulin sensitivity, hepatic steatosis index (HSI), fatty liver index (FLI) and a pro-inflammatory score). Newly emerging risk markers of type 2 diabetes (fetuin A, fibroblast growth factor 21 (FGF-21) [16], interleukin-1 receptor antagonist (IL-1ra) and omentin [17]) were considered as secondary outcomes.

## 2. Methods

### 2.1. DONALD Study

The DONALD (DOrtmund Nutritional and Anthropometric Longitudinally Designed) study is an ongoing, open cohort study conducted in Dortmund, Germany, that was previously described [18]. In brief, since 1985, detailed data on diet, growth, development, and metabolism between infancy and early adulthood have been collected from approximately 1550 healthy children. Each year, 30–35 infants are newly recruited and first examined at the ages of three or six months. Participants regularly return to the study center thereafter; assessment during adolescence is scheduled annually. Since 2005, participants aged 18+ years are requested to provide a fasting blood sample in addition to the regular examination. The study was approved by the Ethics Committee of the University of Bonn (project identification code: 098/06, date of approval 21 June 2006); all examinations are performed with written consent from the parents and/or the participants.

### 2.2. Dietary Assessment

Nutritional data are assessed by 3-day weighed dietary records on three consecutive days. Participants are free to choose the days of recording. Dietary records include information on the timing of meal consumption. Parents and/or participants are instructed by dietitians to weigh all consumed foods and beverages, including leftovers, to the nearest 1 g. To this end, they receive regularly calibrated electronic food scales (initially Soehnle Digita 8000 (Leifheit AG, Nassau, Germany), now WEDO digi 2000 (Werner Dorsch GmbH, Muenster/Dieburg, Germany)). When exact weighing is not possible, semi-quantitative measures (e.g., number of spoons) are allowed. Moreover, information on recipes and on the types and brands of food items consumed is requested. The dietary records are collected as well as reviewed by the dietitians and analyzed using the continuously updated in-house nutrient database LEBTAB [19]. It includes information from standard nutrient tables, product labels, or recipe simulations based on the listed ingredients and nutrients.

### 2.3. Blood Analysis

Venous blood samples are drawn after an overnight fast, centrifuged at 4 °C and frozen at −80 °C in the DONALD Study Center. Fasting plasma glucose was determined on a Roche/Hitachi Cobas c 311 analyzer (Basel, Switzerland). Plasma insulin concentration was measured at the Laboratory for Translational Hormone Analytics of the University of Giessen using an immunoradiometric assay (IRMA, DRG Diagnostics, Marburg, Germany). All other measurements were performed at the German Diabetes Center with assay characteristics as described [5,20,21,22]: plasma activities of alanine-aminotransferase (ALT), aspartate-aminotransferase (AST), gamma-glutamyltransferase (GGT), plasma triglycerides (TG) and plasma high-sensitivity C-reactive protein (hsCRP) with the Roche/Hitachi Cobas c311 analyzer (Roche diagnostics, Mannheim, Germany), plasma high-sensitivity interleukin-(IL)-6 using the Human IL-6 Quantikine HS, plasma adiponectin with the Human Total Adiponectin/Acrp30 Quantikine ELISA, serum leptin with the Leptin Quantikine ELISA, serum interleukin-1 receptor antagonist (IL-1ra) with the Human IL-1ra/IL-1F3 ELISA, plasma fibroblast growth factor (FGF-21) with the Human FGF-21 kits all from R & D Systems (Wiesbaden, Germany), serum IL-18 using the Human IL-18 ELISA kit from MBL (Nagoya, Japan), and plasma chemerin with the Human Chemerin ELISA, plasma omentin-1 with the Human Omentin-1 ELISA, plasma chemerin with the Human Chemerin ELISA and plasma fetuin-A with the Human Fetuin-A ELISA kits from BioVendor (Brno, Czech Republic).

Insulin sensitivity was assessed using the updated HOMA2 sensitivity (in %) based on fasting insulin and blood glucose [23]. Indices of hepatic steatosis were calculated as follows:

Hepatic steatosis index (HSI):

- HSI = 8 × ALT/AST + BMI
  (+2, if female; +2, if diabetes mellitus, but not applied, as the DONALD study excludes participants with chronic disease) [24],
- Fatty liver index (FLI):
  FLI = e*^x^*/(1 + e*^x^*) × 100
  with *x* = 0.953 × ln(TG) + 0.139 × BMI + 0.718 × ln(GGT) + 0.053 × waist circumference − 15.745 [25].

To estimate associations with chronic low-grade inflammation a pro-inflammatory score of established inflammation markers—assumed to be more predictive of inflammation than single markers [26]—was obtained by averaging inflammation markers, that were standardized (*z*) by sex (mean = 0, SD = 1) beforehand. To align anti-inflammatory adiponectin in the pro-inflammatory score it was multiplied by −1:

Pro-inflammatory score = (*z*-hsCRP + *z*-IL-6 + *z*-IL18 + *z*-chemerin + *z*-adiponectin × (−1) + *z*-leptin)/6.

### 2.4. Anthropometric Data

Anthropometric measurements are performed by trained nurses according to standard procedures, with the participants dressed only in underwear and barefoot. Standing height is measured to the nearest 0.1 cm (digital stadiometer: Harpenden Ltd., Crymych, UK) and body weight to the nearest 0.1 kg (electronic scale: model 753 E; Seca, Hamburg, Germany). Waist circumference is measured at the midpoint between the lower rib and the iliac crest to the nearest 0.1 cm. Measurements of skinfold thicknesses are taken on the right side of the body at the biceps, triceps, subscapular and suprailiac sites to the nearest 0.1 mm (Holtain caliper: Holtain Ltd., Crymych, UK).

From these measures, adolescent body mass index (BMI, kg/m^2^) and its sex- and age-specific SD-scores (SDS) were calculated using current German BMI standards [27]. The prevalence of overweight in adolescence was calculated using standards of the International Obesity TaskForce (IOTF) [28]. Percent body fat (%BF) in adolescence and adulthood was estimated using the Slaughter [29] and the Durnin-Womersley equations [30], respectively. From these, body fat mass (kg) was calculated [(%BF× body mass)/100] and related to the square of height to obtain fat mass index (FMI, kg/m^2^).

### 2.5. Family Characteristics

On a child’s admission to the study, information on gestational characteristics and birth anthropometrics are abstracted from a standardized document (Mutterpass), given to all pregnant women in Germany. Moreover, parents are interviewed concerning the child’s early life data as well as family and socio-economic characteristics at regular intervals. Additionally, parents are regularly weighed and measured using the same equipment used for participants.

### 2.6. Definition of Morning and Evening Intake

The definition of morning and evening was described in earlier analyses [31,32]. Briefly, for each age (year), the time between the end of the night and 11 a.m. and the time between 6 p.m. and the start of the night, respectively, was used to define morning and evening.

For estimation of morning and evening GI and GL as well as morning and evening carbohydrate intake from low- or higher-GI food sources, each carbohydrate containing food recorded in the corresponding day-time window was assigned a published GI value [33,34], based on glucose as reference food and according to a standardized procedure [35]. Carbohydrate content of each food consumed in the morning or in the evening was then multiplied by the food’s GI to obtain the GL of the corresponding day-time window. Subsequently, overall morning/evening GI was obtained by dividing morning/evening GL by morning/evening carbohydrate intake. To distinguish carbohydrates from low- and higher-GI food sources, a value of 55 (cutoff proposed for low-GI foods [34]) was chosen for categorization. Accordingly, morning and evening carbohydrate intake from low-GI sources (low-GI-CHO (GI < 55) in g) and from moderate- or high-GI sources (higher-GI-CHO (GI ≥ 55) in g) was calculated.

### 2.7. Study Sample

Due to the open-cohort design of the study, many subjects have not yet reached young adulthood, when blood samples are taken. At the time of this analysis *N* = 414 young adults had provided at least one fasting blood sample, in which risk markers of type 2 diabetes were quantified. As only term (gestational age 37–42 weeks) singletons with a minimum birth weight of 2500 g were included in the analyses, sample size was reduced to *N* = 397. To allow estimation of morning and evening GI, GL, low-GI-CHO and higher-GI-CHO during adolescence (girls: 9–15 years, boys: 10–16 years), at least two 3-day weighed dietary records from three consecutive days were required (*N* = 298). Participants, who were identified to consistently underreport their energy intake were excluded from the study (*N* = 12, resulting in *N* = 286). In line with earlier publications [4,5] ‘consistent underreporting’ was defined to be present, when more than half of the available food records per participant were implausible. A 3-day weighed dietary record was considered implausible, when the total energy intake documented was inadequate in relation to the basal metabolic rate (estimated from Schofield equations [36]) using cut-offs from Goldberg et al. [37] modified for children and adolescents [38]. Finally, anthropometric measurements from adolescence and young adulthood as well as data on relevant covariates were required, resulting in analyses samples of *N* = 253 for liver steatosis outcomes, *N* = 252 for insulin sensitivity and *N* = 249 for inflammation-related outcomes. Sample sizes for insulin sensitivity and inflammation outcomes were lower because of fasting glucose concentrations below the threshold for calculation of HOMA2 sensitivity (reflecting hypoglycemia) or insufficient amounts of blood were available for determination of analytes.

### 2.8. Statistical Analysis

SAS procedures (SAS version 9.2, SAS Institute, Cary, NC, USA) were used for data analysis. *p*-values < 0.05 were considered significant. Tests for interaction indicated no sex differences for the primary outcomes, except for the relevance of evening GL for the pro-inflammatory score. Since stratified analyses for this association yielded similar non-significant results for males and females, all associations are from pooled analyses.

Characteristics of the study population are presented as medians (25th, 75th percentiles) for continuous variables and as absolute (relative) frequencies for categorical variables.

The prospective associations of morning and evening GI, GL, low-GI-CHO and higher-GI-CHO with the risk markers of type 2 diabetes were analyzed using multivariable linear regression models. To achieve normal distribution in the outcome variables, they were transformed prior to analysis using log, double-log, square root or reciprocal transformations, depending on the outcome. In addition, individual outliers which substantially interfered with normal distribution or regression modeling were winsorized, i.e., outliers were replaced by the sex-specifically closest value fitting a normal distribution. The procedure concerned IL-6 (*N* = 4), adiponectin (*N* = 1) and IL-1ra (*N* = 2). Exposures were energy-adjusted for morning and evening energy intake, respectively—except for dietary GI-using the residual method [39]. To account for sex- and age-dependent differences in nutritional intake, all variables were standardized (mean = 0, SD = 1) by sex and age (year) and averaged over the time period of adolescence. Crude models (model A) included the exposure variable as well as sex and age at blood withdrawal in adulthood. In a further step, adjusted models (model B) were constructed by individual examination of potentially influencing covariates and hierarchical inclusion [40] of those which substantially modified the exposure’s regression coefficient by ≥10% [41] or independently predicted the outcome variable [42]. Potential confounding covariates considered for inclusion in the different hierarchical levels were (1) early life characteristics (gestational weight gain (kg), mother’s age at birth (years), duration of pregnancy (weeks) and birth weight (g), first-born child (yes/no), full breastfeeding (≥4 months yes/no)), (2) family and socio-economic characteristics (parental diabetes (yes/no), maternal overweight (≥25 kg/m^2^ yes/no), maternal educational status (≥12 year of schooling yes/no), smoking in the household (yes/no)), (3) baseline characteristics (adolescent BMI-SDS, intake of saturated fatty acids (SFA, E%) and intake of animal protein (E%)). To ensure comparability, models were adjusted identically according to the strongest exposure-outcome associations for closely related outcomes (i.e., for (i) insulin sensitivity, (ii) hepatic steatosis markers (HSI, FLI, fetuin A, FGF-21) and (iii) inflammatory markers (pro-inflammatory score, IL-1ra, omentin)). For significant associations (model B), we additionally ran conditional models including waist circumferences to examine whether the observed associations are partly attributable to effects of carbohydrate nutrition on body composition. As HSI and FLI do by definition already include adult BMI and/or waist circumference, no conditional models were run for these outcomes.

## 3. Results

Characteristics of the study population at baseline and in young adulthood are shown in Table 1 and Table 2, respectively (see Section 2.7. for selection of the sample). During adolescence, the GI and GL as well as the relative contribution of carbohydrates from low- or higher-GI sources were broadly comparable between morning and evening intake. Note that the carbohydrate content of morning intake was higher than the carbohydrate content of evening intake. In young adulthood, median age at blood withdrawal was 21 years (range 18–39 years).

Overall, **morning** GI and GL as well as morning carbohydrate intake from low- or higher-GI sources were not related to insulin sensitivity (Table S1), the primary hepatic steatosis outcomes HSI and FLI (Tables S2 and S3) or the pro-inflammatory score (Table 3, top) in young adulthood (all *p* > 0.1). Of note, low-GI-CHO intake during the morning was related to HSI in model A (*p* = 0.041), however, upon further adjustment this association was no longer statistically significant (Table S2, model B, *p* = 0.24). Morning exposures were neither related to the secondary outcomes fetuin A (Table S4), FGF-21 (Table S5), and IL-1ra (Table S6) (all *p* > 0.08). However, a higher morning GI during adolescence was associated with an increased level of the secondary outcome omentin in young adulthood (*p* = 0.011, Table S7); an association that persisted in the conditional model adjusting for adult waist circumference (*p* = 0.003).

**Figure 1:**
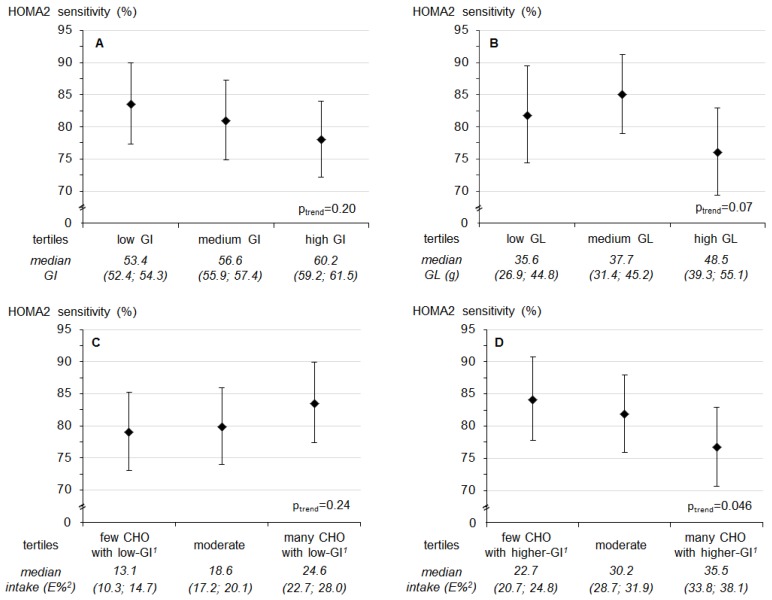
Predicted least square means (95% confidence interval) of **HOMA2 sensitivity** by tertiles of (**A**) glycemic index (GI) after 6 p.m., (**B**) glycemic load (GL) after 6 p.m., (**C**) intake of low-GI-CHO ^1^ after 6 p.m. and (**D**) intake of higher-GI-CHO ^1^ after 6 p.m. Values are least-square means for tertiles obtained from linear regression models. *p*-values are based on models using the continuous exposure variables. Models were adjusted for first born child (yes/no), baseline BMI-SDS, baseline evening intake of saturated fatty acids and animal protein (*N* = 252). Values below the figure refer to median intakes (25th; 75th percentiles) in each tertile of the respective exposure. ^1^ Distinction between carbohydrate intake from low- and higher-GI food sources with a GI of 55 as cut-off; ^2^ % of energy intake after 6 p.m.

**Figure 2:**
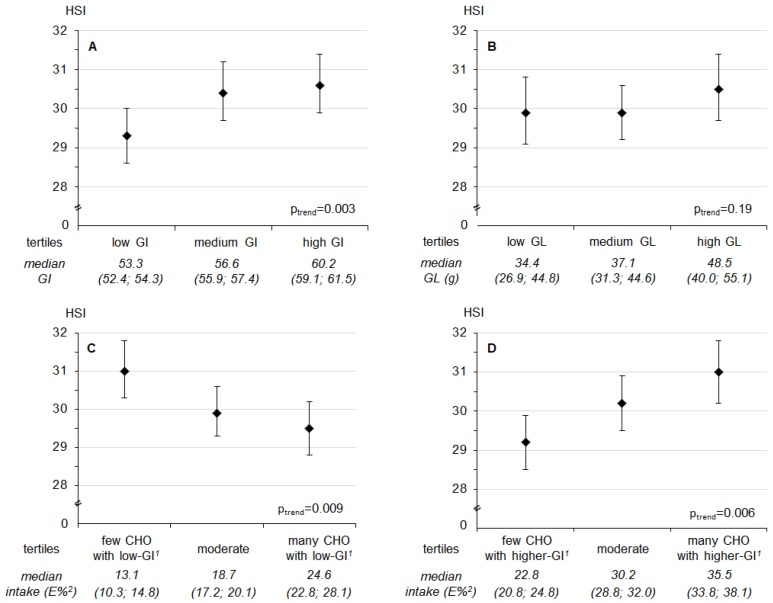
Predicted least square means (95% confidence interval) of **hepatic steatosis index (HSI)** by tertiles of (**A**) glycemic index (GI) after 6 p.m., (**B**) glycemic load (GL) after 6 p.m., (**C**) intake of low-GI-CHO ^1^ after 6 p.m. and (**D**) intake of higher-GI-CHO ^1^ after 6 p.m. Values are least-square means for tertiles obtained from linear regression models. *p*-values are based on models using the continuous exposure variables. Models were adjusted for sex, age at blood withdrawal, gestational weight gain, duration of pregnancy and birth weight, maternal educational status (≥12 years of schooling yes/no), maternal overweight (≥25 kg/m^2^ yes/no), baseline BMI-SDS, and baseline evening intake of saturated fatty acids (*N* = 253). Values below the figure refer to median intakes (25th; 75th percentiles) in each tertile of the respective exposure. ^1^ Distinction between carbohydrate intake from low- and higher-GI food sources with a GI of 55 as cut-off; ^2^ % of energy intake after 6 p.m.

In contrast, a higher **evening** carbohydrate intake from higher-GI sources during adolescence was associated with lower insulin sensitivity in young adulthood (*p* = 0.046, Figure 1). Additional adjustment for waist circumference rendered this association non-significant (*p* =0.11). A higher evening GI (*p* = 0.003) and a higher evening higher-GI-CHO intake (*p* = 0.006) were related to an increased HSI, whereas higher evening low-GI-CHO intake (*p* = 0.009) was associated with a lower HSI (Figure 2). Similarly, a higher evening GL (*p* = 0.005) and higher evening higher-GI-CHO intake (*p* = 0.029) were associated with increased concentrations of the secondary outcome fetuin A (Table S4); neither of these relations was explained by adult waist circumference (*p* = 0.006 and *p* = 0.040 respectively). No prospective associations of evening intakes were observed with the primary outcomes FLI (Table S3) and the pro-inflammatory score (Table 3, bottom), or with the secondary outcomes FGF-21 (Table S5), IL-1ra (Table S6) and omentin (Table S7) (all *p* > 0.01).

## 4. Discussion

The present study provides novel evidence that adverse longer-term metabolic effects of recurring postprandial glycemic excursions may be specific to evening carbohydrate consumption. Specifically, adolescents who habitually consumed more carbohydrates from higher-GI food sources in the evening had a lower insulin sensitivity and a higher HSI in young adulthood. By contrast, such adverse prospective associations were not observed for morning intakes. The absence of associations with the pro-inflammatory score suggests that day-time specific intake pattern in adolescence may not be of longer-term relevance for low-grade inflammation among young, healthy adults.

Our observation linking evening higher-GI-CHO to insulin sensitivity is in line with our hypothesis that evening rather than morning intake of higher-GI foods is potentially detrimental for risk factors of type 2 diabetes. A diurnal pattern of insulin sensitivity has been confirmed for both healthy persons [11] and participants with prediabetes [12]. The observed decrease in insulin sensitivity over the course of the day offers a plausible mechanism for our results. In line with our findings, a recent study measuring 20-h day-time profiles from 6 healthy volunteers (4 females, 2 males) reported that estimated postprandial insulin sensitivity was lowest when participants consumed 60% of their daily energy intake in the form of high-GI foods at supper [43]. In an earlier study from the same group, 8-h daytime profiles were collected from 17 middle-aged men with overweight or obesity and at least one cardiac risk factor [44]. Results revealed higher postprandial glucose and insulin responses following high-GI lunch and afternoon tea, but not high-GI breakfast compared to the corresponding low-GI meals. Upon adherence to the assigned diet over 24 days, postprandial insulin resistance had increased in the high-GI diet group compared to the low-GI diet group; thus, there was no metabolic adaptation to the high-GI diet. Our results expand on these findings in that they suggest that higher-GI-CHO habitually consumed in the evening by adolescents may have longer-term adverse consequences for adult insulin sensitivity. Our conditional model suggests that this association may be partly mediated by adult body composition. This is supported by the fact that lower glycogen synthesis over night was observed in subjects with diabetes mellitus type 2 compared to insulin-sensitive subjects [45], so that high evening higher-GI-CHO intake may shift glucose metabolites to de-novo lipogenesis and subsequent fat storage among adolescents experiencing physiological insulin resistance. Hence, future studies should also address the relevance of higher-GI-CHO consumed in the evening for body composition.

Glucose metabolism is closely linked to fatty acid metabolism; therefore insulin resistance and accumulation of fat in the liver are tightly interrelated [46]. Two studies, although heterogeneous in their methods (intervention vs observational study, extreme (dietary GI 32 vs. 84) vs. habitual low/high-GI diets and determination of liver steatosis severity by ^1^H magnetic resonance spectroscopy and liver ultrasonography scanning, respectively), suggest that the dietary GI may be related to liver function [47,48]. However, it should be noted that imaging methods as used in these studies are time- and cost-intensive and therefore often infeasible in observational studies.

Our study used validated indices, which are preferable over the use of single liver enzyme activities in that they include more than one metabolic parameter predictive of hepatic steatosis. Our results extend existing evidence [47,48] suggesting that a higher-GI diet and an increased intake of higher-GI-CHO are both of longer-term relevance for hepatic steatosis and that these associations are specific to evening intakes.

It is important to note that HSI was shown to reflect the presence as well as the degree of hepatic steatosis [24]. The cut-off value to rule-out hepatic steatosis is 30, while the cut-off value to postulate the presence of hepatic steatosis is 36. In our healthy sample, only those in the lowest tertile of higher-GI-CHO intake had HSI levels clearly below 30, whereas those in the middle and highest tertiles had mean HSI values between 30 and 31 (Figure 2).

Similarly, albeit non-significant associations were observed with the FLI. Both HSI and FLI have similar efficacy to detect steatosis compared to an imaging method and were described as appropriate surrogate markers for epidemiological studies [49]. The two main differences between HSI and FLI are that the latter considers the activity of GGT instead of ALT and AST activities as well as TG and waist circumference in addition to BMI. In an earlier analysis of ours, dietary GI during adolescence had been related to both GGT and ALT in young adulthood [4], but a recent meta-analysis does not support an independent effect of the GI-level of diets on TG concentrations [50]. Therefore, the FLI could by definition be less responsive to the exposures under consideration in the present analysis. Yet, chance must be considered as a possible explanation as well. However, it was noted that results for fetuin A—one of our secondary outcomes, which is closely related to hepatic steatosis [16]—are in line with the findings for HSI and were independent of waist circumference.

We did not observe a relation of morning or evening GI, GL, or intake of low-GI-CHO or higher-GI-CHO to the pro-inflammatory score. In an earlier study of ours, a higher dietary GL as well as higher daily intakes of higher-GI-CHO during adolescence were associated with increased levels of IL-6, but not with hs-CRP, IL-18 or adiponectin in young adulthood [5]. Individual analyses of parameters which are included in the score confirmed these results for the current sample, albeit without an indication of a day-time specificity: Both a higher morning and a higher evening GL as well as higher intakes of morning and evening higher-GI-CHO were associated with increased adult levels of IL-6 (data not shown).

Conversely, a day-time specificity emerged for omentin. Here, higher morning GI during adolescence was associated with increased young adult omentin levels. The association was unaffected by additional adjustment for adult waist circumference. Consistent with our results, data from healthy normal weight children showed higher insulin levels and lower insulin sensitivity in children in the highest as compared to the lowest tertile of omentin [51]. As omentin is discussed to exert anti-inflammatory actions [17], we speculate that our findings may reflect an upregulation due to a habitual counter-regulatory omentin increase in response to regular pro-inflammatory signals. Indeed, a high-GI diet—regularly provoking postprandial glycemic spikes—induces increased oxidative stress [52]. In terms of the day-time specificity, it is worth noting that postprandial glycemic spikes following high GI meals are highest after breakfast compared to lunch and tea times [44].

If confirmed by other studies, our findings have several potential implications: First, the time of day when higher-GI foods are consumed is of relevance, so that a shift in focus is needed from ‘what we eat’ also to ‘when do we eat what’. Second, our observations are based on habitual dietary intake data and reveal that the analyzed population consumed on average more than half of their morning and evening carbohydrates from higher-GI food sources, which translates into a third of their morning and evening energy intake from higher-GI food sources. Of note, those who consumed less than a quarter of their evening energy intake from higher-GI-CHO (i.e., those in the lowest tertile) had a mean HSI below 30. Hence, our data suggest that reducing higher-GI-CHO intake from a third to a quarter of energy intake in the evening offers preventive potential for adult type 2 diabetes risk. Third, the absence of a relation with morning GI, GL, low-GI-CHO or higher-GI-CHO does not justify a recommendation to shift high evening intake of high-GI foods to morning hours, as there is a possibility of adverse longer-term effects for chronic subclinical inflammation in response to habitual postprandial spikes induced in the morning. Moreover, due to the fact that the delay in chronotype (i.e., the preference for a delayed timing of sleep on free days) peaks approximately at the age of 20 years [13], encouraging morning food intake may augment circadian misalignment and would therefore be counterproductive for metabolic health in adolescents and young adults.

Our study is limited by the availability of one blood sample in young adulthood only; the use of surrogate markers of diabetes risk instead of hard end points can be considered as further limitation. However, our population was too young to have established type 2 diabetes. Moreover, it needs to be assumed that the analyzed outcomes themselves follow individual circadian rhythms [53,54] so that measurement of risk markers takes place in blood samples potentially withdrawn during the acrophase (i.e., the maximum value of one rhythm cycle) of one parameter and the bathyphase (i.e., the minimum value of one rhythm cycle) of another parameter. However, withdrawing all blood samples at the approximately same day-time, i.e., during morning hours in our study, results in a standardization of all outcome parameters to day-time. Consequently, differences in the circadian rhythm between the analytes do not affect the calculated indices (HOMA2 sensitivity, HSI, FLI, pro-inflammatory score). Concerning our dietary predictors, the GI concept is still contentious. Recent criticism relates to methodological aspects [55] and GI extrapolation to mixed meals [56]. However, the validity of dietary GI has been demonstrated repeatedly using different methodological approaches [15,57] and ISO-standardization will further reduce methodological errors in measuring the GI of foods [58]. It could be criticized that we excluded only participants who regularly under-reported their energy intake from our analyses. However, exclusion of under-reporters is controversial [59] and it should be noted that in our study only a further *N* = 77 participants had ever underreported their energy intake in any of their protocols (median: 7 protocols). Additional exclusion of these participants did not affect our results. Only the association of higher-GI-CHO with HOMA2 sensitivity was attenuated towards a trend (*p* = 0.08), which is likely attributable to the lower number of participants included in this sensitivity analysis (data not shown). Overall, our results could be subject to concerns about multiple testing. However, three separate sets of primary outcomes were considered (insulin sensitivity, hepatic steatosis, subclinical chronic inflammation) and different mechanisms are discussed for the relevance of GI, GL or CHO from low- or higher-GI sources for these outcomes. Moreover, we abstained from stressing and/or discussing findings that tended to be associated (*p*-values between 0.05 and <0.1). Generally, the DONALD population is characterized by a socio-economic status that is above average [18], so that extremes in nutritional behavior might not be represented. Consequently, a selection bias is likely introduced. However, the relatively homogeneous sample decreases the vulnerability of the results to residual confounding.

The overall strengths of the study are its prospective design and the detailed repeated dietary data including day-time specific nutritional information during adolescence. Due to the recruitment of study participants during infancy and the annually repeated data collection, 3-day weighed dietary records are documented by participants accustomed to the procedure. Moreover, availability of data on several important potential confounders, i.e., early-life characteristics, anthropometrics, familial and socio-economic factors strengthen our analyses.

## 5. Conclusions

In conclusion, our data suggest that young adult insulin sensitivity and hepatic steatosis markers are responsive to higher evening, but not morning intakes of higher-GI-CHO during adolescence, whereas there was no association with adult subclinical inflammation. Avoidance of large amounts of higher-GI-CHO in the evening and/or their replacement by low-GI-CHO during adolescence may present a promising preventive strategy to reduce risk of type 2 diabetes in adulthood.

## Figures and Tables

**Table 1:** Baseline sample characteristics ^1^ (*N* = 252).

**General Characteristics**	
Sex ♀ (*n* (%))	130 (51.6)
Mean age (years)	12.4 (12.0; 13.0)
**Early Life Factors**	
Gestational weight gain (kg)	12 (10; 15)
Mothers age at gestation (years)	30.6 (28.2; 33.3)
Birth weight (g)	3450 (3130; 3810)
Duration of gestation (weeks)	40 (39; 41)
First born child (*n* (%))	152 (60.3)
Fully breastfed, ≥4 months (*n* (%))	124 (49.2)
**Family Characteristics**	
Parental diabetes (*n* (%))	9 (3.6)
Maternal overweight, ≥25 kg/m^2^ (*n* (%))	101 (40.1)
Maternal educational status, ≥12 years of schooling (*n* (%))	136 (54.0)
Smoking in the household (*n* (%))	72 (28.6)
**Body Composition during Adolescence ^2^**	
BMI (kg/m^2^)	18.6 (16.8; 20.2)
BMI Standard Deviation Score	−0.13 (−0.87; 0.37)
FMI (kg/m^2^)	3.2 (2.4; 4.5)
Overweight (*n* (%)) ^3^	31 (12.3)
**Nutrition Parameters during Adolescence ^2^**	
Number of 3-day dietary records per participant	7 (6; 7)
**Daily** energy intake (kcal)	1922 (1658; 2158)
Total carbohydrates (E% ^4^)	51.0 (48.4; 54.3)
Total protein (E% ^4^)	13.0 (12.0; 14.1)
Animal protein (E% ^4^)	8.1 (7.2; 9.1)
Total fat (E% ^4^)	35.9 (32.9; 38.1)
SFA (E% ^4^)	15.7 (14.1; 17.1)
Energy intake **before 11 a.m.** (kcal)	506 (418; 603)
Energy intake **before 11 a.m.** (E% ^4^)	26.7 (22.7; 30.7)
GI	56.2 (53.6; 58.8)
GL (g)	38.0 (32.5; 47.1)
Carbohydrates with low-GI ^5^ (E% ^6^)	21.2 (16.8; 26.6)
Carbohydrates with higher-GI ^5^ (E% ^6^)	32.6 (26.8; 37.3)
Total carbohydrates (E% ^6^)	54.5 (49.9; 59.6)
Total protein (E% ^6^)	12.0 (10.6; 13.6)
Animal protein (E% ^6^)	6.8 (5.3; 8.4)
Total fat (E% ^6^)	32.7 (28.3; 36.3)
SFA (E% ^6^)	15.0 (12.7; 17.6)
Energy intake **after 6 p.m.** (kcal)	580 (471; 691)
Energy intake **after 6 p.m.** (E% ^4^)	30.3 (26.7; 34.1)
GI	56.6 (54.3; 59.2)
GL (g)	40.2 (31.5; 49.1)
Carbohydrates with low-GI ^5^ (E% ^6^)	18.6 (14.7; 23.1)
Carbohydrates with higher-GI ^5^ (E% ^6^)	30.2 (24.8; 34.0)
Total carbohydrates (E% ^6^)	48.7 (44.8; 52.9)
Total protein (E% ^6^)	14.0 (12.3; 15.3)
Animal protein (E% ^6^)	9.0 (7.3; 10.5)
Total fat (E% ^6^)	37.1 (33.5; 40.5)
SFA (E% ^6^)	15.6 (14.3; 17.5)

Values are *n* (%) for categorical variables or medians (25th, 75th percentiles) for continuous variables. ^1^ General characteristics are shown for the sample used for diabetic risk markers only (*N* = 252), since samples used for risk marker of hepatic steatosis (*N* = 253) and inflammation (*N* = 249) are very similar; ^2^ mean over six years (♀: 9–15 years, ♂: 10–16 years); ^3^ includes overweight and obesity as defined by IOTF in Cole 2000 [28]; ^4^ % of daily energy intake; ^5^ distinction between carbohydrate intake from low- and higher-GI food sources with a GI of 55 as cut-off; ^6^ % of energy intake before 11 a.m./after 6 p.m. BMI—body mass index, FMI—fat mass index, GL—glycemic load, GI—glycemic index, SFA—saturated fatty acids.

**Table 2:** Sample characteristics for young adulthood ^1^.

	*N*	Value
**General Characteristic**		
Mean age at blood withdrawal (years)	252	21.0 (18.1; 24.0)
**Lifestyle**		
Alcohol consumption (g) ^2^	197	0.3 (0.0; 6.0)
Smokers (*n* (%))	237	65 (27.4)
**Body composition**		
BMI (kg/m²)	252	22.1 (20.6; 24.6)
FMI (kg/m²)	252	5.7 (3.8; 7.2)
Overweight (*n* (%)) ^3^	252	55 (21.8)
Waist circumference (cm)	252	75.9 (70.8; 80.9)
**Risk markers of type 2 diabetes**		
HOMA2 sensitivity (%)	252	77.1 (61.2; 99.0)
Hepatic steatosis index (HSI)	253	29.8 (27.8; 32.8)
Fatty liver index (FLI)	253	7.3 (4.6; 15.4)
Fetuin A (mg/L)	253	273 (241; 306)
FGF-21 (pg/mL)	253	83.4 (39.7; 156.6)
Pro-inflammatory score	249	−0.11 (−0.38; 0.32)
IL-1ra (pg/mL)	249	218 (169; 295)
Omentin (ng/mL)	249	379 (317; 458)

Values are *n* (%) for categorical variables and median (25th, 75th percentiles) for continuous variables. ^1^ General characteristics are shown for the sample used for diabetic risk markers only (*N* = 252), since samples used for risk marker of hepatic steatosis (*N* = 253) and inflammation (*N* = 249) are very similar; ^2^ estimation is based on *N* = 197 participants who had provided a 3-day weighed dietary record in young adulthood; ^3^ including all BMI ≥ 25.0; BMI—body mass index, FGF-21—*Fibroblast growth factor 21*, FMI—fat mass index, HOMA2—updated homeostasis model assessment for insulin sensitivity, IL-1ra—Interleukin 1 receptor antagonist.

**Table 3:** Prospective relation of GI and GL of morning (before 11 a.m.) and evening (after 6 p.m.) intake as well as morning and evening intake from low- and higher-GI food sources during adolescence to **pro-inflammatory score** in young adulthood (*N* = 249).

	Predicted Means ^1^ of Pro-Inflammatory Score by Exposure Tertiles *(Exposures: Morning and Evening GI, GL, Low-GI-CHO, Higher-GI-CHO)*			*p* for Trend ^2^
	Low Exposure *(T1)*	Average Exposure *(T2)*	High Exposure *(T3)*	
**MORNING**				
**Glycemic Index (GI)**				
*Median GI*	*52.2 (50.2; 53.8)*	*56.2 (55.2; 57.0)*	*59.5 (58.5; 60.6)*	
Model A ^3^	−0.10 (−0.22; 0.03)	−0.03 (−0.15; 0.10)	−0.04 (−0.16; 0.08)	0.15
Model B ^4^	−0.10 (−0.21; 0.02)	−0.04 (−0.16; 0.08)	−0.02 (−0.13; 0.10)	0.18
**Glycemic Load (GL)**				
*Median GL*	*35.7 (30.4; 41.6)*	*35.9 (30.4; 42.9)*	*45.4 (37.1; 53.9)*	
Model A ^3^	−0.08 (−0.20; 0.04)	−0.02 (−0.14; 0.10)	−0.06 (−0.18; 0.06)	0.40
Model B ^4^	−0.11 (−0.23; 0.02)	0.00 (−0.12; 0.12)	−0.05 (−0.17; 0.08)	0.27
**CHO with low-GI** ^5^				
*Median low-GI-CHO (E%)*	*15.4 (12.4; 17.3)*	*21.4 (19.5; 23.3)*	*28.8 (26.5; 32.7)*	
Model A ^3^	0.01 (−0.12; 0.14)	−0.05 (−0.17; 0.08)	−0.13 (−0.25; 0.00)	0.16
Model B ^4^	−0.01 (−0.13; 0.11)	−0.03 (−0.14; 0.09)	−0.11 (−0.23; 0.00)	0.39
**CHO with higher-GI** ^5^				
*Median higher-GI-CHO (E%)*	*25.0 (22.5; 27.2)*	*32.9 (31.0; 34.3)*	*38.6 (36.5; 42.8)*	
Model A ^3^	−0.15 (−0.26; −0.03)	−0.06 (−0.18; 0.07)	0.04 (−0.08; 0.17)	0.06
Model B ^4^	−0.14 (−0.25; −0.02)	−0.04 (−0.16; 0.08)	0.03 (−0.10; 0.15)	0.12
**EVENING**				
**Glycemic Index (GI)**				
*Median GI*	*53.3 (52.4; 54.3)*	*56.6 (55.9; 57.4)*	*60.2 (59.1; 61.5)*	
Model A ^3^	0.04 (−0.09; 0.17)	−0.05 (−0.17; 0.08)	−0.15 (−0.27; −0.03)	0.30
Model B ^4^	0.01 (−0.11; 0.13)	−0.04 (−0.16; 0.08)	−0.12 (−0.23; 0.00)	0.75
**Glycemic Load (GL)**				
*Median GL*	*34.4 (26.9; 44.8)*	*37.1 (31.3; 44.6)*	*48.5 (40.0; 55.1)*	
Model A ^3^	−0.01 (−0.14; 0.12)	−0.17 (−0.29; −0.05)	0.02 (−0.10; 0.15)	0.66
Model B ^4^	−0.05 (−0.18; 0.09)	−0.13 (−0.24; −0.01)	0.03 (−0.10; 0.16)	0.46
**CHO with low-GI** ^5^				
*Median low-GI-CHO (E%)*	*13.1 (10.3; 14.7)*	*18.5 (17.2; 20.0)*	*24.6 (22.8; 28.2)*	
Model A ^3^	−0.07 (−0.19; 0.06)	−0.06 (−0.18; 0.06)	−0.04 (−0.16; 0.09)	0.56
Model B ^4^	−0.05 (−0.17; 0.08)	−0.06 (−0.18; 0.06)	−0.05 (−0.17; 0.07)	0.84
**CHO with higher-GI** ^5^				
*Median higher-GI-CHO (E%)*	*22.5 (20.8; 24.9)*	*30.2 (28.8; 32.0)*	*35.4 (33.7; 38.1)*	
Model A ^3^	−0.09 (−0.21; 0.03)	−0.07 (−0.19; 0.05)	0.00 (−0.12; 0.13)	0.69
Model B ^4^	−0.11 (−0.23; 0.01)	−0.06 (−0.17; 0.06)	0.02 (−0.10; 0.14)	0.44

*Values in italic refer to median intakes (25th, 75th percentiles) in each tertile of the respective exposure.* ^1^ Model-values are least square means (95% confidence intervals) of pro-inflammatory score; ^2^ *p*-values for models are based on linear regression analyses using continuous exposure variables; ^3^ Model A (crude model) adjusted for sex and age at blood withdrawal; ^4^ Model B additionally adjusted for gestational weight gain, maternal educational status (≥12 years of schooling yes/no), baseline BMI-SDS, baseline morning or evening intake of animal protein; ^5^ Distinction between carbohydrate intake from low- and higher-GI food sources with a GI of 55 as cut-off.

## Supplementary Materials

The following are available online at www.mdpi.com/2072-6643/9/6/591/s1. Table S1: Prospective relation of GI and GL of morning (before 11 a.m.) intake as well as morning carbohydrate intake from low- and higher-GI food sources during adolescence to HOMA2 sensitivity in young adulthood (*N* = 252), Table S2: Prospective relation of GI and GL of morning (before 11 a.m.) intake as well as morning carbohydrate intake from low- and higher-GI food sources during adolescence to hepatic steatosis index (HSI) in young adulthood (*N* = 253), Table S3: Prospective relation of GI and GL of morning (before 11 a.m.) and evening (after 6 p.m.) intake as well as morning and evening intake from low- and higher-GI food sources during adolescence to fatty liver index (FLI) in young adulthood (*N* = 253), Table S4: Prospective relation of GI and GL of morning (before 11 a.m.) and evening (after 6 p.m.) intake as well as morning and evening intake from low- and higher-GI food sources during adolescence to fetuin A (mg/L) in young adulthood (*N* = 253), Table S5: Prospective relation of GI and GL of morning (before 11 a.m.) and evening (after 6 p.m.) intake as well as morning and evening intake from low- and higher-GI food sources during adolescence to fibroblast growth factor 21 (FGF-21, pg/mL) in young adulthood (*N* = 253), Table S6: Prospective relation of GI and GL of morning (before 11 a.m.) and evening (after 6 p.m.) intake as well as morning and evening intake from low- and higher-GI food sources during adolescence to interleukin 1 receptor antagonist (IL-1ra, pg/mL) in young adulthood (*N* = 249), Table S7: Prospective relation of GI and GL of morning (before 11 a.m.) and evening (after 6 p.m.) intake as well as morning and evening intake from low- and higher-GI food sources during adolescence to omentin (ng/mL) in young adulthood (*N* = 249).
