# Supplementary material for: Carbohydrates from Sources with a Higher Glycemic Index during Adolescence: Is Evening Rather than Morning Intake Relevant for Risk Markers of Type 2 Diabetes in Young Adulthood?
Source: Nutrients. 2017 Jun 10;9(6):591. doi: 10.3390/nu9060591 (PMC5490570; doi:10.3390/nu9060591)
Supplement: Supplementary file 1 [file nutrients-09-00591-s001.pdf]

SUPPLEMENTARY MATERIAL

Article

CARBOHYDRATES FROM SOURCES WITH A HIGHER GLYCEMIC INDEX DURING ADOLESCENCE: IS EVENING RATHER THAN MORNING INTAKE RELEVANT FOR RISK MARKERS OF TYPE 2 DIABETES IN YOUNGER ADULTHOOD?

Tanja Diederichs, Christian Herder, Sarah Roßbach, Michael Roden, Stefan A Wudy, Ute Nöthlings, Ute Alexy and Anette E Buyken

SUMMARY of supplemental material

Table S1: Prospective relation of GI and GL of morning (before 11 am) intake as well as morning carbohydrate intake from low- and higher-GI food sources during adolescence to **HOMA2 sensitivity** in young adulthood (N=252).

Table S2: Prospective relation of GI and GL of morning (before 11 am) intake as well as morning carbohydrate intake from low- and higher-GI food sources during adolescence to **hepatic steatosis index (HSI)** in young adulthood (N=253).

Table S3: Prospective relation of GI and GL of morning (before 11 am) and evening (after 6 pm) intake as well as morning and evening intake from low- and higher-GI food sources during adolescence to **fatty liver index (FLI)** in young adulthood (N=253).

Table S4: Prospective relation of GI and GL of morning (before 11 am) and evening (after 6 pm) intake as well as morning and evening intake from low- and higher-GI food sources during adolescence to **fetuin A (mg/l)** in young adulthood (N=253).

Table S5: Prospective relation of GI and GL of morning (before 11 am) and evening (after 6 pm) intake as well as morning and evening intake from low- and higher-GI food sources during adolescence to **fibroblast growth factor 21 (FGF-21, pg/ml)** in young adulthood (N=253).

Table S6: Prospective relation of GI and GL of morning (before 11 am) and evening (after 6 pm) intake as well as morning and evening intake from low- and higher-GI food sources during adolescence to **interleukin 1 receptor antagonist (IL-1ra, pg/ml)** in young adulthood (N=249).

Table S7: Prospective relation of GI and GL of morning (before 11 am) and evening (after 6 pm) intake as well as morning and evening intake from low- and higher-GI food sources during adolescence to **omentin (ng/ml)** in young adulthood (N=249).

Table S1: Prospective relation of GI and GL of morning (before 11 am) intake as well as morning intake from low- and higher-GI food sources during adolescence to **HOMA2 sensitivity (%)** in young adulthood (N=252).

	**Predicted means [1] of HOMA2 sensitivity (%) by exposure tertiles** *(exposures: morning GI, GL, low-GI-CHO, higher-GI-CHO)*			**p for trend [2]**
	**low exposure** *(T1)*	**average exposure** *(T2)*	**high exposure** *(T3)*	
		**MORNING**		
**Glycemic index (GI)**				
Median GI	*52.2 (50.0; 53.8)*	*56.3 (55.2; 57.0)*	*59.6 (58.7; 60.8)*	
Model A [3]	81.2 (75.2; 87.4)	80.2 (74.3; 86.3)	79.2 (73.4; 85.3)	0.78
Model B [4]	80.9 (75.0; 87.1)	81.4 (75.5; 87.5)	79.8 (74.0; 85.8)	0.95
**Glycemic load (GL)**				
Median GL	*35.7 (30.4; 42.7)*	*36.1 (30.5; 43.0)*	*45.1 (36.8; 53.9)*	
Model A [3]	75.5 (69.8; 81.4)	81.1 (75.3; 87.1)	84.0 (78.1; 90.2)	0.23
Model B [4]	76.6 (69.4; 84.1)	81.5 (75.7; 87.5)	84.0 (76.9; 91.5)	0.96
**CHO with low-GI [5]**				
Median low-GI-CHO (E%)	*15.4 (12.4; 17.1)*	*21.3 (19.5; 23.3)*	*29.0 (26.6; 33.0)*	
Model A [3]	78.7 (72.8; 84.8)	79.0 (73.2; 85.0)	83.0 (77.0; 89.2)	0.27
Model B [4]	80.7 (74.7; 86.9)	80.1 (74.3; 86.1)	81.4 (75.3; 87.6)	0.90
**CHO with higher-GI [5]**				
Median higher-GI-CHO (E%)	*25.0 (22.5; 27.2)*	*32.9 (31.0; 34.3)*	*39.5 (36.7; 42.8)*	
Model A [3]	79.7 (73.8; 85.8)	81.0 (75.1; 87.1)	79.9 (74.0; 86.0)	0.83
Model B [4]	80.4 (74.4; 86.6)	82.3 (76.3; 88.5)	79.5 (73.4; 85.8)	0.99

Values in italic refer to median intakes (25th, 75th percentile) in each tertile of the respective exposure.
[1] Model-values are least square means (95% confidence intervals) of HOMA2 sensitivity;
[2] P-values for models are based on linear regression analyses using continuous exposure variables;
[3] Model A (crude model) adjusted for sex and age at blood withdrawal;
[4] Model B additionally adjusted for first born child (yes/no), baseline BMI-SDS, baseline morning intake of saturated fatty acids and animal protein;
[5] Distinction between carbohydrate intake from low- and higher-GI food sources with a GI of 55 as cut-off.

Table S2: Prospective relation of GI and GL of morning (before 11 am) intake as well as morning carbohydrate intake from low- and higher-GI food sources during adolescence to **hepatic steatosis index (HSI)** in young adulthood (N=253).

	**Predicted means [1] of HSI by exposure tertiles** *(exposures: morning GI, GL, low-GI-CHO, higher-GI-CHO)*			**p for trend [2]**
	**low exposure** *(T1)*	**average exposure** *(T2)*	**high exposure** *(T3)*	
		**MORNING**		
**Glycemic index (GI)**				
Median GI	*52.2 (50.1; 53.8)*	*56.2 (55.2; 57.0)*	*59.6 (58.7; 60.8)*	
Model A [3]	29.6 (28.8; 30.5)	30.5 (31.5; 29.6)	30.0 (29.1; 30.9)	0.43
Model B [4]	29.6 (28.9; 30.3)	30.5 (29.8; 31.3)	30.3 (29.6; 31.0)	0.42
**Glycemic load (GL)**				
Median GL	*35.7 (30.4; 41.8)*	*36.1 (30.7; 43.0)*	*45.1 (36.8; 53.7)*	
Model A [3]	30.6 (29.7; 31.6)	30.2 (29.4; 31.2)	29.3 (28.5; 30.2)	0.44
Model B [4]	30.3 (29.5; 31.2)	30.5 (29.8; 31.3)	29.5 (28.7; 30.3)	0.94
**CHO with low-GI [5]**				
Median low-GI-CHO (E%)	*15.4 (12.5; 17.3)*	*21.3 (19.5; 23.3)*	*29.0 (26.6; 33.0)*	
Model A [3]	31.0 (30.0; 31.9)	29.8 (28.9; 30.7)	29.4 (28.6; 30.3)	0.041
Model B [4]	30.7 (30.0; 31.5)	30.0 (29.3; 30.7)	29.6 (28.9; 30.4)	0.24
**CHO with higher-GI [5]**				
Median higher-GI-CHO (E%)	*25.0 (22.6; 27.2)*	*32.9 (31.0; 34.3)*	*38.9 (36.6; 42.7)*	
Model A [3]	29.6 (28.8; 30.5)	30.0 (29.1; 30.9)	30.6 (29.7; 31.5)	0.27
Model B [4]	29.7 (29.0; 30.4)	30.3 (29.6; 31.0)	30.4 (29.6; 31.1)	0.37

Values in italic refer to median intakes (25th, 75th percentile) in each tertile of the respective exposure.
[1] Model-values are least square means (95% confidence intervals) of the HSI;
[2] P-values for models are based on linear regression analyses using continuous exposure variables;
[3] Model A (crude model) adjusted for sex and age at blood withdrawal;
[4] Model B additionally adjusted for gestational weight gain, duration of pregnancy and birth weight, maternal educational status (≥12 years of schooling yes/no), maternal overweight (≥25kg/m² yes/no), baseline BMI-SDS, baseline morning intake of saturated fatty acids;
[5] Distinction between carbohydrate intake from low- and higher-GI food sources with a GI of 55 as cut-off.

Table S3: Prospective relation of GI and GL of morning (before 11 am) and evening (after 6 pm) intake as well as morning and evening intake from low- and higher-GI food sources during adolescence to **fatty liver index (FLI)** in young adulthood (N=253).

	**Predicted means [1] of FLI by exposure tertiles** *(exposures: morning and evening GI, GL, low-GI-CHO, higher-GI-CHO)*			**p for trend [2]**
	**low exposure** *(T1)*	**average exposure** *(T2)*	**high exposure** *(T3)*	
		**MORNING**		
**Glycemic index (GI)**				
Median GI	*52.2 (50.1; 53.8)*	*56.2 (55.2; 57.0)*	*59.6 (58.7; 60.8)*	
Model A [3]	7.9 (6.7; 9.5)	8.4 (7.1; 10.1)	7.6 (6.4; 9.1)	0.85
Model B [4]	7.9 (6.8; 9.1)	8.4 (7.3; 9.8)	8.0 (7.0; 9.3)	0.90
**Glycemic load (GL)**				
Median GL	*35.7 (30.4; 41.8)*	*36.1 (30.7; 43.0)*	*45.1 (36.8; 53.7)*	
Model A [3]	8.5 (7.1; 10.2)	8.4 (7.1; 10.1)	7.1 (6.0; 8.5)	0.47
Model B [4]	8.0 (6.8; 9.5)	8.9 (7.7; 10.4)	7.4 (6.4; 8.8)	0.85
**CHO with low-GI [5]**				
Median low-GI-CHO (E%)	*15.4 (12.5; 17.3)*	*21.3 (19.5; 23.3)*	*29.0 (26.6; 33.0)*	
Model A [3]	9.3 (7.8; 11.3)	7.8 (6.6; 9.3)	7.0 (5.9; 8.3)	0.08
Model B [4]	9.0 (7.7; 10.5)	8.2 (7.1; 9.5)	7.2 (6.2; 8.4)	0.34
**CHO with higher-GI [5]**				
Median higher-GI-CHO (E%)	*25.0 (22.6; 27.2)*	*32.9 (31.0; 34.3)*	*38.9 (36.6; 42.7)*	
Model A [3]	7.4 (6.3; 8.9)	7.5 (6.4; 8.9)	9.1 (7.6; 11.0)	0.30
Model B [4]	7.6 (6.6; 8.8)	8.0 (7.0; 9.3)	8.7 (7.5; 10.2)	0.48
		**EVENING**		
**Glycemic index (GI)**				
Median GI	*53.4 (52.4; 54.3)*	*56.6 (55.9; 57.4)*	*60.2 (59.2; 61.5)*	
Model A [3]	8.9 (7.5; 10.7)	8.5 (7.1; 10.1)	6.8 (5.8; 8.0)	0.08
Model B [4]	7.9 (6.8; 9.2)	9.0 (7.8; 10.5)	7.5 (6.5; 8.6)	0.98
**Glycemic load (GL)**				
Median GL	*35.6 (26.9; 44.8)*	*37.0 (31.4; 45.2)*	*48.5 (39.9; 55.1)*	
Model A [3]	8.3 (7.0; 10.0)	7.1 (6.0; 8.4)	8.6 (7.3; 10.4)	0.62
Model B [4]	7.9 (6.7; 9.4)	7.7 (6.7; 9.0)	8.7 (7.4; 10.3)	0.49
**CHO with low-GI [5]**				
Median low-GI-CHO (E%)	*13.1 (10.3; 14.8)*	*18.7 (17.2; 20.1)*	*24.6 (22.8; 28.1)*	
Model A [3]	7.9 (6.6; 9.4)	8.1 (6.9; 9.7)	7.9 (6.7; 9.5)	0.58
Model B [4]	8.7 (7.5; 10.2)	8.1 (7.1; 9.4)	7.5 (6.5; 8.7)	0.36
**CHO with higher-GI [5]**				
Median higher-GI-CHO (E%)	*22.8 (20.8; 24.8)*	*30.2 (28.8; 32.0)*	*35.5 (33.8; 38.1)*	
Model A [3]	7.5 (6.3; 8.9)	8.0 (6.7; 9.5)	8.5 (7.2; 10.3)	0.58
Model B [4]	7.0 (6.1; 8.1)	8.4 (7.3; 9.8)	9.0 (7.7; 10.6)	0.15

Values in italic refer to median intakes (25th, 75th percentile) in each tertile of the respective exposure.

[1] Model-values are least square means (95% confidence intervals) of the FLI;
[2] P-values for models are based on linear regression analyses using continuous exposure variables;
[3] Model A (crude model) adjusted for sex and age at blood withdrawal;
[4] Model B additionally adjusted for gestational weight gain, duration of pregnancy and birth weight, maternal educational status (≥12 years of schooling yes/no), maternal overweight (≥25kg/m² yes/no), baseline BMI-SDS, baseline morning or evening intake of saturated fatty acids;
[5] Distinction between carbohydrate intake from low- and higher-GI food sources with a GI of 55 as cut-off.

Table S4: Prospective relation of GI and GL of morning (before 11 am) and evening (after 6 pm) intake as well as morning and evening intake from low- and higher-GI food sources during adolescence to **fetuin A (mg/l)** in young adulthood (N=253).

	**Predicted means [1] of fetuin A (mg/l) by exposure tertiles** *(exposures: morning and evening GI, GL, low-GI-CHO, higher-GI-CHO)*			**p for trend [2]**
	**low exposure** *(T1)*	**average exposure** *(T2)*	**high exposure** *(T3)*	
		**MORNING**		
**Glycemic index (GI)**				
Median GI	*52.2 (50.1; 53.8)*	*56.2 (55.2; 57.0)*	*59.6 (58.7; 60.8)*	
Model A [3]	273 (263; 284)	270 (260; 280)	278 (267; 288)	0.53
Model B [4]	276 (266; 286)	269 (259; 279)	278 (268; 289)	0.77
**Glycemic load (GL)**				
Median GL	*35.7 (30.4; 41.8)*	*36.1 (30.7; 43.0)*	*45.1 (36.8; 53.7)*	
Model A [3]	273 (263; 283)	268.9 (259.0; 279.0)	279 (268; 289)	0.87
Model B [4]	269 (258; 281)	270.0 (260.1; 280.2)	284 (273; 296)	0.60
**CHO with low-GI [5]**				
Median low-GI-CHO (E%)	*15.4 (12.5; 17.3)*	*21.3 (19.5; 23.3)*	*29.0 (26.6; 33.0)*	
Model A [3]	273 (263; 283)	274.8 (264.8; 285.1)	273 (263; 283)	0.83
Model B [4]	274 (263; 284)	274.3 (264.2; 284.7)	275 (264; 286)	0.75
**CHO with higher-GI [5]**				
Median higher-GI-CHO (E%)	*25.0 (22.6; 27.2)*	*32.9 (31.0; 34.3)*	*38.9 (36.6; 42.7)*	
Model A [3]	275 (265; 285)	270 (260; 280)	276 (266; 286)	0.82
Model B [4]	276 (265; 286)	270 (260; 280)	277 (267; 288)	0.92
		**EVENING**		
**Glycemic index (GI)**				
Median GI	*53.4 (52.4; 54.3)*	*56.6 (55.9; 57.4)*	*60.2 (59.2; 61.5)*	
Model A [3]	270 (260; 280)	275 (265; 286)	275 (265; 285)	0.91
Model B [4]	271 (261; 282)	276 (266; 287)	275 (265; 285)	0.74
**Glycemic load (GL)**				
Median GL	*35.6 (26.9; 44.8)*	*37.0 (31.4; 45.2)*	*48.5 (39.9; 55.1)*	
Model A [3]	266 (256; 276)	267 (260; 276)	288 (278; 299)	0.026
Model B [4]	262 (251; 273)	268 (258; 278)	293 (281; 304)	0.005
**CHO with low-GI [5]**				
Median low-GI-CHO (E%)	*13.1 (10.3; 14.8)*	*18.7 (17.2; 20.1)*	*24.6 (22.8; 28.1)*	
Model A [3]	274 (264; 284)	272 (262; 282)	274 (264; 284)	0.72
Model B [4]	275 (265; 286)	273 (263; 283)	275 (264; 285)	0.74
**CHO with higher-GI [5]**				
Median higher-GI-CHO (E%)	*22.8 (20.8; 24.8)*	*30.2 (28.8; 32.0)*	*35.5 (33.8; 38.1)*	
Model A [3]	265 (255; 275)	274 (264; 284)	282 (272; 292)	0.039
Model B [4]	265 (254; 275)	274 (264; 285)	283 (273; 294)	0.029

Values in italic refer to median intakes (25th, 75th percentile) in each tertile of the respective exposure.
[1] Model-values are least square means (95% confidence intervals) of the fetuin A;
[2] P-values for models are based on linear regression analyses using continuous exposure variables;
[3] Model A (crude model) adjusted for sex and age at blood withdrawal;
[4] Model B additionally adjusted for gestational weight gain, duration of pregnancy and birth weight, maternal educational status (≥12 years of schooling yes/no), maternal overweight (≥25kg/m² yes/no), baseline BMI-SDS, baseline morning or evening intake of saturated fatty acids;
[5] Distinction between carbohydrate intake from low- and higher-GI food sources with a GI of 55 as cut-off.

Table S5: Prospective relation of GI and GL of morning (before 11 am) and evening (after 6 pm) intake as well as morning and evening intake from low- and higher-GI food sources during adolescence to **fibroblast growth factor 21 (FGF-21, pg/ml)** in young adulthood (N=253).

	**Predicted means [1] of FGF-21 (pg/ml) by exposure tertiles** *(exposures: morning and evening GI, GL, low-GI-CHO, higher-GI-CHO)*			**p for trend [2]**
	**low exposure** *(T1)*	**average exposure** *(T2)*	**high exposure** *(T3)*	
		**MORNING**		
**Glycemic index (GI)**				
Median GI	*52.2 (50.1; 53.8)*	*56.2 (55.2; 57.0)*	*59.6 (58.7; 60.8)*	
Model A [3]	73.5 (59.3; 90.7)	71.9 (58.2; 88.7)	93.4 (75.8; 114.7)	0.09
Model B [4]	73.9 (59.3; 91.7)	71.5 (57.5; 88.6)	94.2 (76.2; 116.2)	0.09
**Glycemic load (GL)**				
Median GL	*35.7 (30.4; 41.8)*	*36.1 (30.7; 43.0)*	*45.1 (36.8; 53.7)*	
Model A [3]	73.3 (59.3; 90.5)	78.0 (63.2; 96.0)	86.3 (69.9; 106.2)	0.57
Model B [4]	64.6 (50.2; 82.8)	79.7 (64.3; 98.4)	96.8 (76.1; 122.7)	0.12
**CHO with low-GI [5]**				
Median low-GI-CHO (E%)	*15.4 (12.5; 17.3)*	*21.3 (19.5; 23.3)*	*29.0 (26.6; 33.0)*	
Model A [3]	85.3 (68.8; 105.4)	73.0 (59.0; 90.0)	79.4 (64.1; 97.9)	0.46
Model B [4]	85.1 (68.2; 105.9)	72.4 (58.2; 89.7)	80.8 (64.3; 101.0)	0.58
**CHO with higher-GI [5]**				
Median higher-GI-CHO (E%)	*25.0 (22.6; 27.2)*	*32.9 (31.0; 34.3)*	*38.9 (36.6; 42.7)*	
Model A [3]	67.6 (54.6; 83.4)	91.6 (74.4; 112.5)	79.4 (64.3; 97.9)	0.97
Model B [4]	66.5 (53.2; 82.8)	90.8 (73.1; 112.3)	82.1 (65.8; 102.1)	0.24
		**EVENING**		
**Glycemic index (GI)**				
Median GI	*53.4 (52.4; 54.3)*	*56.6 (55.9; 57.4)*	*60.2 (59.2; 61.5)*	
Model A [3]	71.3 (57.6; 88.0)	77.9 (63.1; 95.8)	88.9 (72.0; 109.4)	0.22
Model B [4]	70.6 (56.3; 88.1)	77.0 (62.0; 95.3)	91.0 (73.3; 112.7)	0.16
**Glycemic load (GL)**				
Median GL	*35.6 (26.9; 44.8)*	*37.0 (31.4; 45.2)*	*48.5 (39.9; 55.1)*	
Model A [3]	77.8 (62.8; 96.2)	76.0 (61.4; 93.7)	83.5 (67.6; 103.0)	0.24
Model B [4]	80.6 (62.8; 103.0)	76.3 (61.2; 94.8)	80.6 (63.4; 102.0)	0.34
**CHO with low-GI [5]**				
Median low-GI-CHO (E%)	*13.1 (10.3; 14.8)*	*18.7 (17.2; 20.1)*	*24.6 (22.8; 28.1)*	
Model A [3]	81.4 (65.8; 100.4)	77.9 (63.1; 96.0)	77.8 (62.9; 96.1)	0.74
Model B [4]	84.3 (67.3; 105.1)	78.0 (63.0; 96.3)	75.7 (60.6; 94.3)	0.42
**CHO with higher-GI [5]**				
Median higher-GI-CHO (E%)	*22.8 (20.8; 24.8)*	*30.2 (28.8; 32.0)*	*35.5 (33.8; 38.1)*	
Model A [3]	72.3 (58.4; 89.3)	80.1 (64.8; 98.6)	85.2 (69.0; 104.9)	0.25
Model B [4]	71.9 (57.2; 90.1)	80.4 (64.8; 99.3)	85.5 (68.3; 106.8)	0.27

Values in italic refer to median intakes (25th, 75th percentile) in each tertile of the respective exposure.
[1] Model-values are least square means (95% confidence intervals) of the FGF-21;
[2] P-values for models are based on linear regression analyses using continuous exposure variables;
[3] Model A (crude model) adjusted for sex and age at blood withdrawal;
[4] Model B additionally adjusted for gestational weight gain, duration of pregnancy and birth weight, maternal educational status (≥12 years of schooling yes/no), maternal overweight (≥25kg/m² yes/no), baseline BMI-SDS, baseline morning or evening intake of saturated fatty acids;
[5] Distinction between carbohydrate intake from low- and higher-GI food sources with a GI of 55 as cut-off.

Table S6: Prospective relation of GI and GL of morning (before 11 am) and evening (after 6 pm) intake as well as morning and evening intake from low- and higher-GI food sources during adolescence to **interleukin 1 receptor antagonist (IL-1ra, pg/ml)** in young adulthood (N=249).

	**Predicted means [1] of IL-1ra (pg/ml) by exposure tertiles** *(exposures: morning and evening GI, GL, low-GI-CHO, higher-GI-CHO)*			**p for trend [2]**
	**low exposure** *(T1)*	**average exposure** *(T2)*	**high exposure** *(T3)*	
		**MORNING**		
**Glycemic index (GI)**				
Median GI	*52.2 (50.2; 53.8)*	*56.2 (55.2; 57.0)*	*59.5 (58.5; 60.6)*	
Model A [3]	205 (189; 223)	208 (192; 226)	212 (196; 232)	0.51
Model B [4]	206 (190; 224)	206 (191; 225)	215 (198; 235)	0.62
**Glycemic load (GL)**				
Median GL	*35.7 (30.4; 41.6)*	*35.9 (30.4; 42.9)*	*45.4 (37.1; 53.9)*	
Model A [3]	202 (187; 220)	210 (194; 229)	213 (196; 233)	0.23
Model B [4]	198 (182; 216)	212 (196; 231)	218 (200; 240)	0.08
**CHO with low-GI [5]**				
Median low-GI-CHO (E%)	*15.4 (12.4; 17.3)*	*21.4 (19.5; 23.3)*	*28.8 (26.5; 32.7)*	
Model A [3]	215 (198; 236)	206 (190; 224)	204 (189; 222)	0.58
Model B [4]	213 (196; 233)	208 (192; 226)	207 (191; 225)	0.96
**CHO with higher-GI [5]**				
Median higher-GI-CHO (E%)	*25.0 (22.5; 27.2)*	*32.9 (31.0; 34.3)*	*38.6 (36.5; 42.8)*	
Model A [3]	205 (190; 224)	201 (186; 218)	220 (202; 241)	0.18
Model B [4]	206 (190; 225)	201 (187; 219)	221 (202; 242)	0.21
		**EVENING**		
**Glycemic index (GI)**				
Median GI	*53.3 (52.4; 54.3)*	*56.6 (55.9; 57.4)*	*60.2 (59.1; 61.5)*	
Model A [3]	216 (199; 236)	216 (199; 236)	194 (180; 211)	0.19
Model B [4]	216 (198; 236)	217 (200; 237)	196 (182; 212)	0.23
**Glycemic load (GL)**				
Median GL	*34.4 (26.9; 44.8)*	*37.1 (31.3; 44.6)*	*48.5 (40.0; 55.1)*	
Model A [3]	231 (211; 252)	193 (180; 209)	204 (189; 222)	0.36
Model B [4]	233 (182; 213)	196 (182; 213)	202 (186; 221)	0.37
**CHO with low-GI [5]**				
Median low-GI-CHO (E%)	*13.1 (10.3; 14.7)*	*18.5 (17.2; 20.0)*	*24.6 (22.8; 28.2)*	
Model A [3]	204 (189; 222)	210 (194; 229)	211 (194; 230)	0.57
Model B [4]	204 (188; 222)	211 (195; 230)	213 (196; 233)	0.41
**CHO with higher-GI [5]**				
Median higher-GI-CHO (E%)	*22.5 (20.8; 24.9)*	*30.2 (28.8; 32.0)*	*35.4 (33.7; 38.1)*	
Model A [3]	211 (230; 194)	206 (225; 191)	208 (227; 192)	0.39
Model B [4]	212 (233; 195)	207 (226; 192)	208 (227; 192)	0.34

Values in italic refer to median intakes (25th, 75th percentile) in each tertile of the respective exposure.
[1] Model-values are least square means (95% confidence intervals) of IL-1ra;
[2] P-values for models are based on linear regression analyses using continuous exposure variables;
[3] Model A (crude model) adjusted for sex and age at blood withdrawal;
[4] Model B additionally adjusted for gestational weight gain, maternal educational status (≥12 years of schooling yes/no), baseline BMI-SDS, baseline morning or evening intake of animal protein;
[5] Distinction between carbohydrate intake from low- and higher-GI food sources with a GI of 55 as cut-off.

Table S7: Prospective relation of GI and GL of morning (before 11 am) and evening (after 6 pm) intake as well as morning and evening intake from low- and higher-GI food sources during adolescence to **omentin (ng/ml)** in young adulthood (N=249)

	**Predicted means [1] for omentin (ng/ml) by exposure tertiles** *(exposures: morning and evening GI, GL, low-GI-CHO, higher-GI-CHO)*			**p for trend [2]**
	**low exposure** *(T1)*	**average exposure** *(T2)*	**high exposure** *(T3)*	
		**MORNING**		
**Glycemic index (GI)**				
Median GI	*52.2 (50.2; 53.8)*	*56.2 (55.2; 57.0)*	*59.5 (58.5; 60.6)*	
Model A [3]	364 (343; 386)	373 (352; 396)	401 (378; 426)	0.011
Model B [4]	362 (341; 384)	375 (354; 398)	400 (377; 424)	0.003
**Glycemic load (GL)**				
Median GL	*35.7 (30.4; 41.6)*	*35.9 (30.4; 42.9)*	*45.4 (37.1; 53.9)*	
Model A [3]	379 (357; 403)	370 (349; 393)	389 (366; 413)	0.32
Model B [4]	374 (351; 399)	370 (349; 392)	393 (369; 418)	0.09
**CHO with low-GI [5]**				
Median low-GI-CHO (E%)	*15.4 (12.4; 17.3)*	*21.4 (19.5; 23.3)*	*28.8 (26.5; 32.7)*	
Model A [3]	393 (370; 418)	368 (347; 391)	377 (355; 400)	0.29
Model B [4]	397 (373; 422)	366 (345; 388)	375 (353; 398)	0.13
**CHO with higher-GI [5]**				
Median higher-GI-CHO (E%)	*25.0 (22.5; 27.2)*	*32.9 (31.0; 34.3)*	*38.6 (36.5; 42.8)*	
Model A [3]	371 (350; 394)	380 (358; 403)	386 (364; 411)	0.41
Model B [4]	366 (344; 389)	377 (356; 400)	394 (370; 419)	0.10
		**EVENING**		
**Glycemic index (GI)**				
Median GI	*53.3 (52.4; 54.3)*	*56.6 (55.9; 57.4)*	*60.2 (59.1; 61.5)*	
Model A [3]	367 (346; 390)	379 (357; 402)	392 (370; 417)	0.13
Model B [4]	370 (346; 391)	379 (357; 402)	390 (367; 414)	0.21
**Glycemic load (GL)**				
Median GL	*34.4 (26.9; 44.8)*	*37.1 (31.3; 44.6)*	*48.5 (40.0; 55.1)*	
Model A [3]	370 (348; 393)	393 (370; 417)	375 (354; 398)	0.96
Model B [4]	371 (348; 396)	388 (366; 412)	377 (354; 402)	0.97
**CHO with low-GI [5]**				
Median low-GI-CHO (E%)	*13.1 (10.3; 14.7)*	*18.5 (17.2; 20.0)*	*24.6 (22.8; 28.2)*	
Model A [3]	385 (363; 409)	386 (364; 410)	367 (346; 389)	0.23
Model B [4]	384 (361; 408)	386 (364; 409)	367 (346; 390)	0.26
**CHO with higher-GI [5]**				
Median higher-GI-CHO (E%)	*22.5 (20.8; 24.9)*	*30.2 (28.8; 32.0)*	*35.4 (33.7; 38.1)*	
Model A [3]	379 (357; 402)	389 (364; 410)	373 (351; 396)	0.66
Model B [4]	379 (356; 404)	385 (363; 409)	372 (351; 396)	0.64

Values in italic refer to median intakes (25th, 75th percentile) in each tertile of the respective exposure.
[1] Model-values are least square means (95% confidence intervals) of omentin;
[2] P-values for models are based on linear regression analyses using continuous exposure variables;
[3] Model A (crude model) adjusted for sex and age at blood withdrawal;
[4] Model B additionally adjusted for gestational weight gain, maternal educational status (≥12 years of schooling yes/no), baseline BMI-SDS, baseline morning or evening intake of animal protein;
[5] Distinction between carbohydrate intake from low- and higher-GI food sources with a GI of 55 as cut-off.
